# Soil Respiration under Different Land Uses in Eastern China

**DOI:** 10.1371/journal.pone.0124198

**Published:** 2015-04-13

**Authors:** Li-Chao Fan, Ming-Zhen Yang, Wen-Yan Han

**Affiliations:** 1 Tea Research Institute, Chinese Academy of Agricultural Sciences, Hangzhou, China; 2 Graduate School of Chinese Academy of Agricultural Sciences, Beijing, China; University of Maryland, UNITED STATES

## Abstract

Land-use change has a crucial influence on soil respiration, which further affects soil nutrient availability and carbon stock. We monitored soil respiration rates under different land-use types (tea gardens with three production levels, adjacent woodland, and a vegetable field) in Eastern China at weekly intervals over a year using the dynamic closed chamber method. The relationship between soil respiration and environmental factors was also evaluated. The soil respiration rate exhibited a remarkable single peak that was highest in July/August and lowest in January. The annual cumulative respiration flux increased by 25.6% and 20.9% in the tea garden with high production (HP) and the vegetable field (VF), respectively, relative to woodland (WL). However, no significant differences were observed between tea gardens with medium production (MP), low production (LP), WL, and VF. Soil respiration rates were significantly and positively correlated with organic carbon, total nitrogen, and available phosphorous content. Each site displayed a significant exponential relationship between soil respiration and soil temperature measured at 5 cm depth, which explained 84–98% of the variation in soil respiration. The model with a combination of soil temperature and moisture was better at predicting the temporal variation of soil respiration rate than the single temperature model for all sites. Q_10_ was 2.40, 2.00, and 1.86–1.98 for VF, WL, and tea gardens, respectively, indicating that converting WL to VF increased and converting to tea gardens decreased the sensitivity of soil respiration to temperature. The equation of the multiple linear regression showed that identical factors, including soil organic carbon (SOC), soil water content (SWC), pH, and water soluble aluminum (WSAl), drove the changes in soil respiration and Q_10_ after conversion of land use. Temporal variations of soil respiration were mainly controlled by soil temperature, whereas spatial variations were influenced by SOC, SWC, pH, and WSAl.

## Introduction

Terrestrial ecosystems play an important role in the global carbon cycle and are the most active carbon pools that affect the atmospheric CO_2_ concentration [1]. Land-atmosphere CO_2_ exchange is mostly mediated by soil respiration and its slight alteration would lead to a considerable change in the atmospheric CO_2_ concentration [2]. Human activities, as an important driving force of climate change, can alter the magnitude and variability of carbon fluxes in terrestrial ecosystems [3], [4]. Land-use change is one of the human activities that has a significant impact on the variability in terrestrial ecosystems and soil CO_2_ emission, and thus contributes greatly to the increase of atmospheric CO_2_ [5]. However, previous studies concerning the effect of land-use change on soil respiration show strong discrepancies. For instance, Wang *et al*. [6] reported that the annual soil respiration increased by 3–22% after the conversion of grassland to woodland. In another study, Zhang *et al*. [7] observed that the cumulative CO_2_ emission decreased from 45.4 to 34.7 t hm^-2^ a^-1^ when paddy fields were converted to bamboo stands. Liu *et al*. [8] demonstrated that the conversion of a natural broadleaf evergreen forest to a conventionally managed bamboo forest had no significant effect on the total annual soil CO_2_ efflux. These contradictory results require further study to provide clear evidence based on accurate measurements to deepen our current understanding of the effects of land-use change on terrestrial ecosystems and the carbon cycle.

Land-use change is associated with changes in vegetation type and management practices. The changes in vegetation can alter above-ground biomass, soil organic matter content, soil microbial communities, and the plant growth microenvironment, and thus affect soil respiration and temperature fluctuations [9]. Monitoring soil respiration and temperature is therefore a key prerequisite to provide insight into the terrestrial carbon balance [9]. Management practices, such as tillage [10], [11], irrigation [12], and fertilization [13], can alter soil properties and soil organic carbon content, which play important roles in soil carbon storage and soil respiration.

Furthermore, soil respiration is strongly influenced by soil temperature and many studies have shown that soil respiration and temperature exhibit a significant exponential relationship [14], [15], [16], [17]. The Q_10_ value is commonly used to describe the temperature sensitivity of soil respiration. Studies have shown Q_10_ values ranging from 1.3 to 3.3 [18], and different ecological systems have different values [9], [19]. Soil moisture content affects the soil respiration rate mainly through altering soil permeability, circulation of water soluble organic matter, soil structure, and soil microbial activity [20], [21]. However, some research has suggested that the relationship between soil respiration and soil moisture is similar to that between soil respiration and temperature [22], soil moisture even overshadowed temperature control over soil CO_2_ efflux [23], whereas others have found no significant correlation between soil respiration and soil moisture [7], [24].

Tea (*Camellia sinensis* (L.) *O*. *Kuntze*) is a perennial evergreen plant, mainly distributed over tropical and subtropical areas including China, India, Sri Lanka, and Kenya. It is a major cash crop in many developing countries. In recent years, tea cultivation has been expanded rapidly because of its high economic value. The new tea gardens have mainly been converted from woodland or cultivated farmland. However, the effects of land-use change on soil respiration are largely unknown. In this study, we monitored soil respiration for a period of 13 months in five adjacent fields including tea gardens with high, medium, and low production levels; woodland; and a vegetable field. The tea gardens and the vegetable field were converted from the woodland. Our main objectives were (1) to measure annual soil respiration under different land-use types, and (2) to study the major drivers of the variation of soil respiration. Our study provides novel insight into the determiners of soil respiration, which should be taken into consideration in view of global warming when converting woodland to tea gardens or vegetable fields.

## Materials and Methods

### Ethics statement

The administration of the Tea Research Institute, Chinese Academy of Agricultural Sciences provided legal permission to conduct this research at each study site. We confirm that the field studies did not involve endangered or protected species.

### Study area and experimental sites

**Fig 1:**
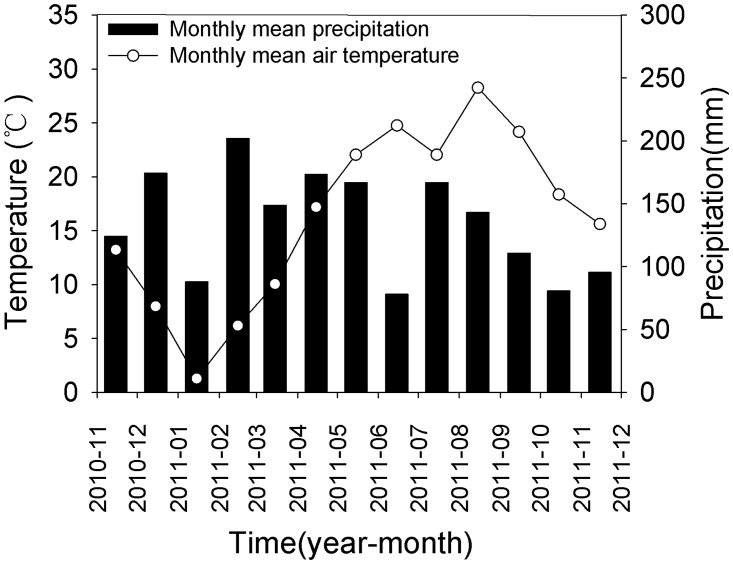
Seasonal mean precipitation and air temperature during the experimental period.

The present study was conducted at the Tea Research Institute, Chinese Academy of Agricultural Sciences (TRI CAAS), Hangzhou, China. The study area is located in Zhejiang Province, Eastern China (120°09’E, 30°14’N), which is famous for producing the Westlake *Longjing* tea. The experimental sites are characterized by a subtropical monsoon climate with four distinct seasons. The annual mean air temperature is 17.0°C with monthly mean air temperatures ranging from 1.7°C in January to 33.0°C in July. The mean annual precipitation is 1533 mm. The monthly mean air temperature and precipitation during the study period are shown in Fig 1.

We selected five fields for our experiment with different land-use types: tea gardens with high production (HP), medium production (MP), and low production (LP), woodland (WL), and a vegetable field (VF). The spatial distance between the study sites is less than 1 km. The basis of classification of these tea gardens is mainly tea yield and fertilizer input, which have been described previously by Han *et al*. [25]. In brief, HP, MP, and LP represent tea yields of 280, 200, and 150 kg hm^-2^ a^-1^, respectively, from spring harvest during mid-March to mid-April, followed by heavy pruning of about 13.4, 9.4, and 6.2 t hm^-2^ a^-1^, respectively, that is left *in situ* as surface mulch. Chemical fertilizers (mainly urea) were applied at approximately 600, 450, and 300 kg N hm^-2^ a^-1^, and organic fertilizer (farmyard manure or rape seed cake) was applied at 2250, 1120, and 1120 kg hm^-2^ a^-1^ during the last few years at the HP, MP, and LP sites, respectively. Other field management was similar and the stand age of the tea plants was approximately 30 years. Woodland was dominated by evergreen broad-leaf plants including *Schima crenata korthals*, *Castanopsis sclerophylla*, and *Cinnamonum camphora*, receiving no fertilizer except litter at approximately 10 t hm^-2^ a^-1^. The vegetable field was covered predominantly by Chinese cabbage *(Brassica rapa chinensis)* and radish *(Raphanus sativus)* throughout the year, receiving approximately 300 kg N hm^-2^ a^-1^ (mainly urea and compound fertilizer), 10 t hm^-2^ a^-1^ organic fertilizer (farmyard manure and biogas slurry), and 4 t hm^-2^ a^-1^ vegetable residues. The tea gardens and vegetable field were converted from woodland in the 1980s. All soil is loamy clay with Anshan quartzfree porphyry as parent material and is classified as *Ultisols* [26]. The basic physicochemical characteristics of the soils are shown in Table 1.

**Table 1:** Basic physicochemical properties of soil at the experimental sites.

Type of land-use	pH (H_2_O, 1:1)	SBD (g cm^-3^)	Total N (g kg^-1^)	Organic C (g kg^-1^)	C/N	Available P (mg kg^-1^)	Available K (mg kg^-1^)	Water soluble Al (mg kg^-1^)	Soil temperature (°C)	Soil water content (%)
**HP**	3.35±0.02 e	1.09	2.8±0.12 a	26.1±0.35 b	10.0±0.25 b	176.4±5.5 b	151.8±3.9 b	1163.9±33.8 a	15.28±2.28a	31.58±1.07b
**MP**	3.64±0.02 c	1.10	2.0±0.11 c	15.0±0.20 d	8.1±0.21 c	39.9±0.8 c	150.2±3.1 b	1135.1±32.5 a	15.63±2.32a	24.08±0.64c
**LP**	3.95±0.03 b	1.18	1.4±0.04 d	12.4±0.25 e	8.7±0.24 c	41.0±0.9 c	121.8±2.3 c	927.5±26.9 c	15.45±2.52a	20.86±0.76d
**WL**	3.53±0.02 d	1.21	1.9±0.04 c	18.4±0.28 c	10.4±0.25 b	31.3±0.7 c	88.3±1.4 d	1045.7±27.2 b	15.09±2.20a	36.39±1.58a
**VF**	6.68±0.03 a	1.05	2.4±0.03 b	29.5±0.32 a	12.6±0.33 a	285.2±7.3 a	245.5±7.9 a	846.3±8.6 d	15.76±2.52a	21.84±1.03cd

HP = tea garden with high production; MP = tea garden with medium production; LP = tea garden with low production; WL = woodland; VF = vegetable field; SBD = soil bulk density. Values are the mean ± standard error (SE),the different lowercase letter in the same column denote significant difference at p<0.05.

### Experimental design and measurement of soil respiration rate

We set up a plot (20 m × 20 m) with five nonadjacent replicated subplots (5 m × 5 m) in each experimental site, and one random measurement point was selected in each subplot. Soil respiration was measured once a week from November 2010 to November 2011 using the dynamic closed chamber method with a portable infrared gas analyzer (GXH-3051A, Beijing JUN FANG Institute, China) that comes with a pump for ventilating and connecting with an autonomous polyvinyl chloride (PVC) chamber (inner diameter: 10.4 cm, height: 12 cm). One PVC collar (inner diameter: 10.4 cm, height: 5 cm) was randomly inserted into 3 cm soil depth in each subplot, and green plants and litter inside the collars at the soil surface were removed one day before measurement. Measurements were taken between 9:00–12:00 am on the same day. The total time at each measurement point was 3 min, and the readings (ppm) were manually recorded at 15 s intervals, with 15 s of dead time between stopping one measurement and starting the next one. Soil temperatures at the surface and at 5 cm depth were measured simultaneously by a handheld long-stem thermometer, and the soil close to the measurement point was sampled to determine soil water content using the oven dry method (105±2°C, 24h). At least five soil augers were used for each measurement point at all sites.

### Soil sampling and measurement

In each field, three replicate samples were measured between 0–15 cm depth using a soil auger, and each sample was homogenized with at least ten points from each subplot to form a composite sample. All soil samples were transported to the laboratory, air-dried, and then passed through a 1 mm sieve for a monthly physicochemical analysis. Soil bulk density was determined using the cutting-ring method and the soil pH using a combined glass electrode in 1:1 (W: V) ratio of soil: distilled water. Total soil N and organic C concentrations were determined with a Vario Max CN Analyzer (Elementar Analysensysteme GmbH, Germany). Available soil P was extracted with 0.03 mol L^-1^ ammonium fluoride (NH_4_F) + 0.025 mol L^-1^ hydrochloric acid (HCl); available soil K was extracted with 1 mol L^-1^ ammonium acetate (NH_4_OAC) and water soluble Al with 0.2 mol L^-1^ calcium chloride (CaCl_2_); and all extractions applied a 1:10 soil-solution ratio and oscillation for 30 min. The elements in the solutions were determined using inductively coupled plasma atomic emission spectroscopy (ICP-AES; JAC IRIS/AP, Thermo Jarrell Ash Corporation, Franklin, USA).

### Data analysis

We used regression analysis to examine the relationship between soil respiration and soil temperature, soil moisture, and the temperature-moisture combination. The equations are as follows.

For temperature:

$$y=a\times e^{b\times T}$$ Equation 1

The temperature sensitivity of the respiration rate (Q_10_) was then calculated by:

$$Q_{10}=e^{10\times b}$$ Equation 2

For soil moisture:

Simple equation:

y=a+bW Equation 3

[27]

Quadratic equation:

$$y=a+bW+cW^{2}$$ Equation 4

[28]

For the soil temperature-moisture combination:

Linear model:

y=a+bT+cW Equation 5

[29]

Exponential model:

ln y=a+bT+cW Equation 6

[30] where y is the soil respiration rate (mg CO_2_-C m^-2^ h^-1^), T is the soil temperature (°C), W is soil moisture (%), and a, b and c are constant coefficients.

We conducted a multiple linear regression (Backward method) using SPSS software (ver.19.0, SPSS Inc., Chicago, IL, USA) to identify the factors responsible for changes in the annual mean soil respiration rate and the Q_10_ value following the conversion of land use. For this analysis, we considered the annual mean soil respiration rate as the dependent variable, and several potential environmental factors as indenpendent variables, such as annual mean soil temperture, annual mean soil water content (SWC), soil organic carbon (SOC), soil total nitrogen, pH, available P, available K, and water soluble Al (WSAl). The annual mean respiration rate was estimated based on the weekly soil respiration rate, and the annual cumulative respiration flux was calculated using the annual mean respiration rate. We used the weekly average value of the five subplots in each site to process the regression analysis between the soil respiration rate and soil temperature, and used monthly data for regression analysis between the soil respiration rate and soil moisture, the temperature-moisture combination, and soil physicochemical variables. A one-way analysis of variation (ANOVA) and the least significant difference (LSD) test were used to determine the statistical significance of the annual mean soil temperature, annual mean soil respiration rate, and some basic soil physicochemical properties at *p*<0.05. SPSS software (ver.19.0, SPSS Inc., Chicago, IL, USA) was used for all statistical analysis. Figures were prepared using sigmaplot version 12.5 software.

## Results

### Soil properties

**Fig 2:**
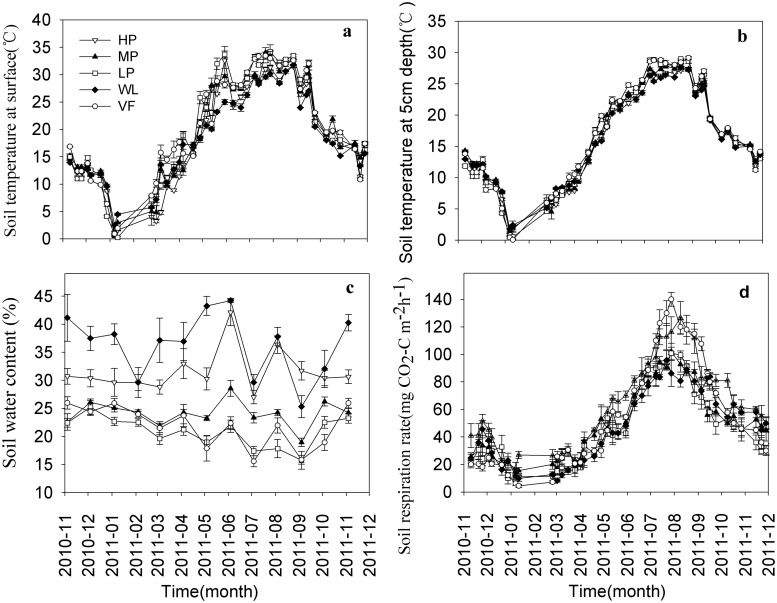
Seasonal change of soil temperature measured (a) at the surface and (b) at 5 cm depth, (c) soil water content in the 0–15 cm soil layer, (d) soil respiration rate at the five experimental sites. HP = tea garden with high production, MP = tea garden with medium production, LP = tea garden with low production, WL = woodland, VF = vegetable field. Vertical bars indicate standard error.

**Fig 3:**
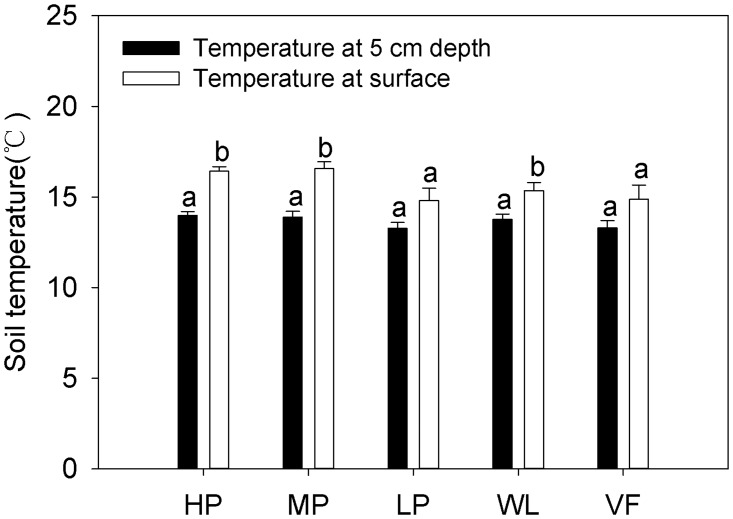
Annual mean soil temperature measured at the surface and 5 cm depth at five adjacent experimental sites. HP = tea garden with high production, MP = tea garden with medium production, LP = tea garden with low production, WL = woodland, VF = vegetable field. Vertical bars indicate standard error. Bars labelled with the different lowercase letters at the same site denote significant difference at *p*<0.05.

We observed no significant difference in soil temperature between the tested sites (Fig 2a and 2b). However, the annual average soil temperature at the surface was higher than that at 5 cm depth (Fig 3) at all sites, and significant difference was found between HP, MP, and WL. Soil water content was highest at WL, followed by HP, MP, VF, and LP (Fig 2c). Soil pH was highest at VF, lowest at HP, and significant differences were observed between the five experimental sites. The soil organic C content was signifcantly different between the five sites, and was highest at VF, followed by HP and WL, and lowest at LP. The total soil N content was highest at HP, followed by VF, MP, and WL, and lowest at LP. Significant differences were found between HP, VF, MP, and LP. Total soil nitrogen content at MP was almost the same as at WL. Available P and K showed large differences and followed the same trend with highest values at VF, and lowest at WL (Table 1).

### Seasonal soil respiration

**Fig 4:**
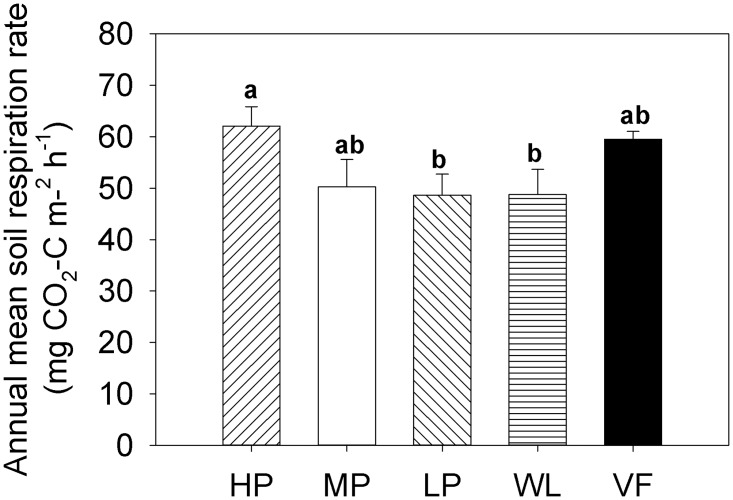
Annual mean soil respiration efflux in five adjacent experimental sites. HP = tea garden with high production, MP = tea garden with medium production, LP = tea garden with low production, WL = woodland, VF = vegetable field. Vertical bars indicate standard error. Bars labelled with the different lowercase letters at the same site denote significant difference at *p*<0.05.

The seasonal variation of the soil respiration rate was consistent with the seasonal variation of temperature and showed a single peak at all sites (Fig 2d). The highest soil respiration rate was recorded in July/August, and the lowest in January. Each site had a high coefficient of variation (CV): 53.9%, 52.7%, 62.6%, 59.9%, and 77.8% in HP, MP, LP, WL, and VF, respectively. The annual mean respiration rate at HP was significantly higher than that at LP or WL, and there was no significant difference between HP, MP, and VF, or between MP, LP, WL, and VF (Fig 4). The annual mean respiration rates were 62.0, 50.3, 48.6, 48.8, and 59.5 mg CO_2_-C m^-2^ h^-1^ at HP, MP, LP, WL, and VF, repectively. The annual cumulative respiration fluxes were 5.4, 4.4, 4.3, 4.3, and 5.2 t CO_2_-C hm^-2^ a^-1^ at HP, MP, LP, WL, and VF, respectively. These fluxes increased by 25.6% and 20.9% when WL was converted to HP and VF, respectively, but showed only small differences when WL was converted to MP and LP.

### Relationship of soil respiration with soil physicochemical characteristics

**Fig 5:**
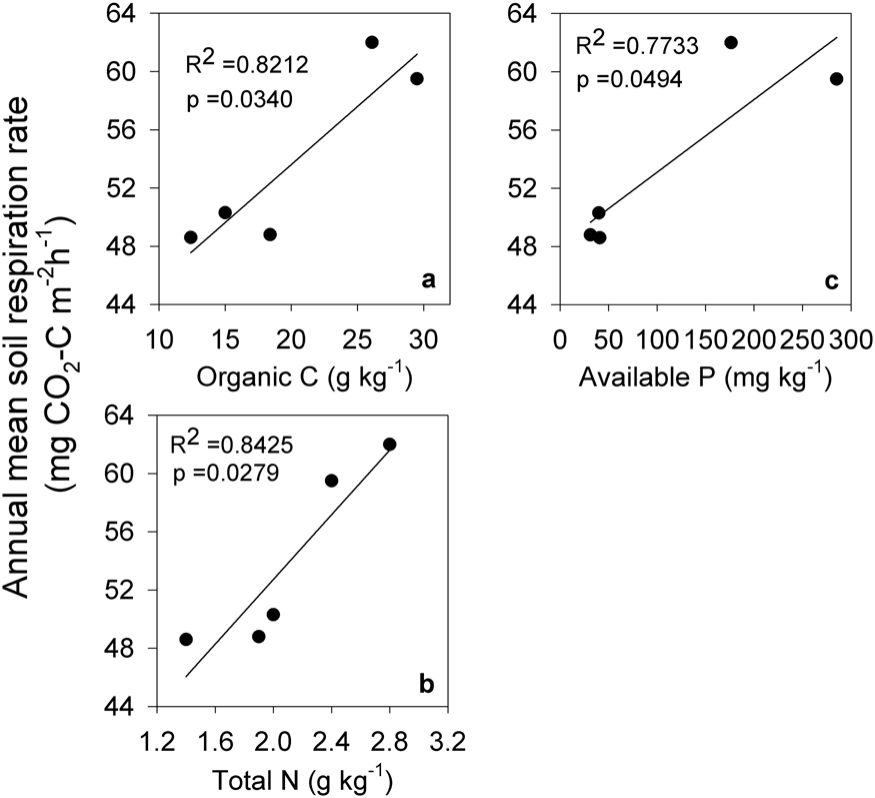
Relationship of annual mean soil respiration rate with organic C, total N, and available P content between the five experimental sites. The data points (n = 5) are the yearly average between the five different experimental sites.

The annual mean respiration rate was predominantly correlated with annual mean organic C, total N, and available P concentrations, and accounted for 82%, 84%, and 77%, respectively, of the variation of annual mean respiration rate at each site (Fig 5). However, there was no significant correlation between the annual mean respiration rate and the C/N ratio (*p* = 0.30), pH (*p* = 0.52), available K (*p* = 0.21), or water soluble Al (*p* = 0.95).

### Relationship of soil respiration with temperature and soil moisture

**Fig 6:**
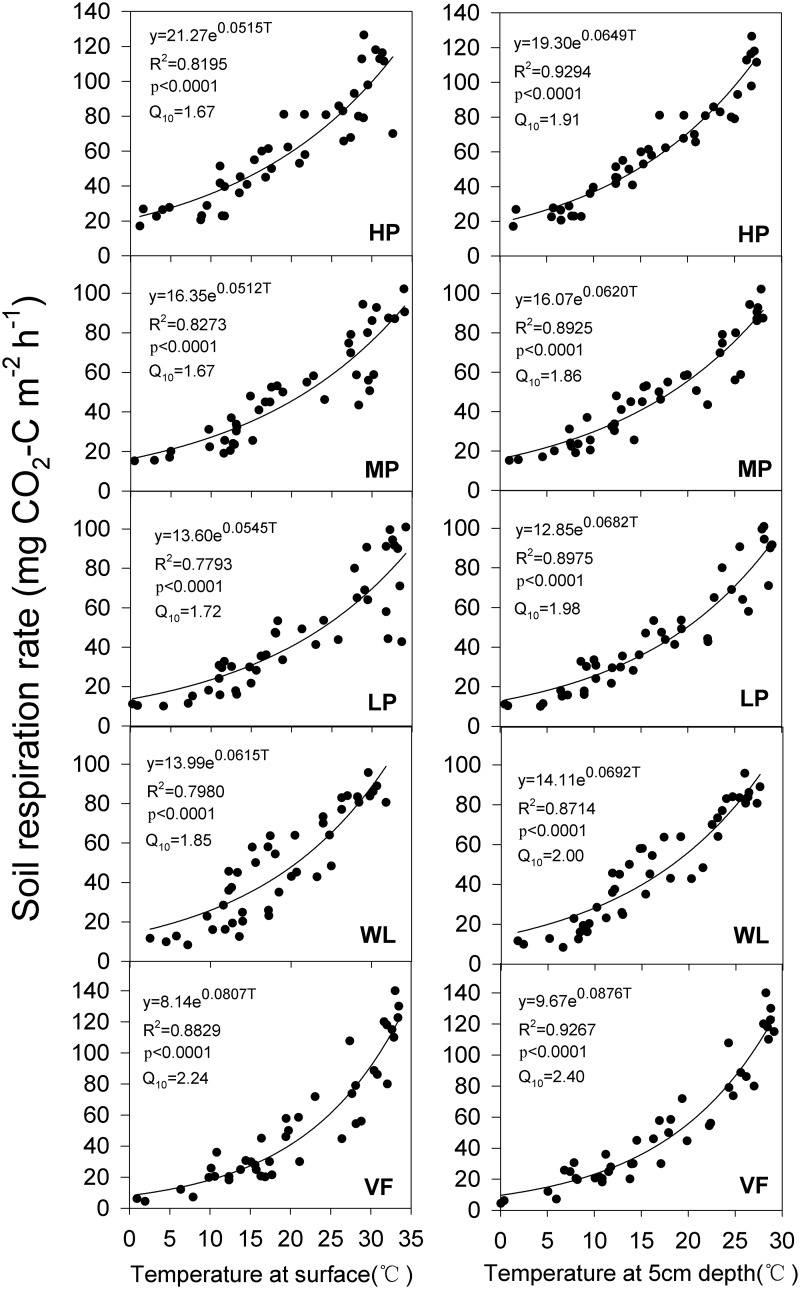
Relationship between the seasonal variation of soil respiration and soil temperature measured at the surface (left) and 5 cm depth (right). HP = tea garden with high production, MP = tea garden with medium production, LP = tea garden with low production, WL = woodland, VF = vegetable field. The data points (n = 43) are the weekly mean of five subplots from each site.

There was a significant exponential relationship between the seasonal variation of soil respiration and temperature at the soil surface and at 5 cm soil depth (Fig 6). The values of R^2^ (coefficient of determination) were higher at 5 cm soil depth than at the soil surface at each site, and the Q_10_ value had the same trend. The Q_10_ value at HP was higher than that at MP, but lower than that at LP (Fig 6). Furthermore, the Q_10_ values decreased when the woodland was converted to tea gardens, and increased when woodland was converted to the vegetable field.

Soil water content was relatively stable over the study period compared with soil temperature. The CV of soil moisture was 12.2%, 9.6%, 13.1%, 15.6%, and 17.0% at HP, MP, LP, WL, and VF, respectively. The *p* values of correlation between soil respiration and soil moisture simulated using Eq 3 were 0.19, 0.91, 0.08, 0.53, and 0.06, and those simulated by Eq 4 were 0.37, 0.66, 0.10, 0.28, and 0.06 at HP, MP, LP, WL, and VF, repectively. Notably, all values were below the level of significance (*p* < 0.05).

Conversely, soil respiration was strongly correlated with the combination of temperature and moisture when simulated using Eqs 5 and 6. Furthermore, the R^2^ value of Eq 6 was higher than that of Eq 5, and explained 84–98% of the variation of the annual soil respiration rate (Table 2).

**Table 2:** Regression equation between the soil respiration rate and soil temperature-moisture.

Type of land-use	Regression equation					
	Linear model Eq 5			Exponential model Eq 6		
**HP**	y = 4.97–0.12W + 3.81T	R^2^ = 0.9396	p<0.0001	lny = 2.92–0.0008W + 0.0684T	R^2^ = 0.9757	p<0.0001
**MP**	y = -26.70 + 1.19W + 2.96T	R^2^ = 0.9341	p<0.0001	lny = 2.22 + 0.0190W + 0.0672T	R^2^ = 0.9565	p<0.0001
**LP**	y = - 31.24 + 1.19W + 3.26T	R^2^ = 0.9345	p<0.0001	lny = 0.81 + 0.0641W + 0.0915T	R^2^ = 0.9820	p<0.0001
**WL**	y = 15.76–0.54W + 3.29T	R^2^ = 0.9687	p<0.0001	lny = 2.24–0.0011W + 0.0880T	R^2^ = 0.9000	p<0.0001
**VF**	y = - 39.09 + 1.02W + 4.60T	R^2^ = 0.8274	P = 0.0002	lny = 1.72 + 0.0097W + 0.1074T	R^2^ = 0.8448	p<0.0001

Note:HP = tea garden with high production; MP = tea garden with medium production; LP = tea garden with low production; WL = woodland; VF = vegetable field.

### Factors influencing soil respiration and Q_10_ under different land uses

Based on the results of the “Backward” regression, four independent variables (SWC, SOC, pH, and WSAl) were chosen for the final equation, while the others were ignored. The final equation was therefore as follows: the annual mean soil respiration rate (Rs) = 72.695–0.62 (SWC) + 1.408 (SOC)- 4.959 (pH)- 0.009 (WSAl). Next, we set the Q_10_ value as the dependent variable, and obtained the final equation as Q_10_ = 1.757 + 0.008 (SWC) + 0.003 (SOC) + 0.122 (pH) + 0.0005 (WSAl). The annual mean soil respiration rate and Q_10_ were therefore driven by identical factors (SWC, SOC, pH, and WSAl) following land use conversion.

## Discussion

### Effects of land-use change on soil properties and soil respiration

Land-use change has a strong effect on soil properties, especially in pool sizes of organic C and total N [31]. In the current study, we found that soil organic C and total N were significantly different between all sites, apart from total N between MP and WL. The annual mean soil respiration rate was significantly correlated with organic C and total N between the experimental sites (Fig 5a and 5b). The results were consistent with the report of Wang *et al*. [6], who found that the annual mean respiration rate was mainly dependent on organic C and total N content in woodland, which was converted from grassland. Organic matter is the substrate of soil respiration, and environmental factors, such as soil temperature and soil moisture, are mainly dependent on the energy that is liberated from the decomposition of soil organic matter [32], [33].

However, the annual cumulative respiration flux was not always consistent with the organic C content. In our study, the annual cumulative respiration flux increased by 25.6% and 20.9% when WL was converted to HP and VF, respectively, but no difference was observed when WL was converted to MP and LP. Organic C was highest at VF, followed by HP (Table 1). Organic C content at MP and LP decreased by 18.5% and 32.6%, respectively, compared with WL (Table 1). In a similar study, Liu *et al*. [8] found that soil respiration efflux and organic C exhibited no significant difference when natural evergreen forest was converted to bamboo forest, but soil respiration efflux increased significantly because of intensive management and organic C decreased from 2.2% to 1.9%. Wang *et al*. [34] studied the effect of land-use change on soil respiration of forest converted to cropland, and found that soil respiration efflux decreased significantly from 5.2 to 3.5 t C hm^-2^ a^-1^, and organic matter also decreased considerably from 19.3 to 12.0 g kg^-1^. These studies suggest that the annual cumulative respiration flux is not always consistent with organic C content as an assignable factor that would change soil structure (e.g., porosity and aggregate structure) [35], and soil microbial community, function, and biomass [36], potentially because of field management. A change in soil structure is more likely to circulate nutrients and oxygen into the soil, and could affect the growth of soil microorganisms and the decomposition of organic matter [37]. An augmentation in soil microbial biomass would increase the rate of organic matter decomposition, and thus enhance soil respiration. In some cases, a change in soil structure may easily lead to organic C erosion [38]. All these changes can reduce the organic C content of soil. Conversely, organic fertilization and litterfall replenish soil organic C [39], and the soil respiration rate increases with increasing organic fertilization [40], [41]. Vegetation types can alter the soil respiration rate by controlling the quantity of litterfall [9], distribution of photoassimilates [42], root biomass [43], soil nutrition, and soil microbial communities [44].

In addition, we found that available P and the annual mean soil respiration rate at the five experimental sites showed significant correlation (Fig 5c). Similarly, Zhou *et al*. [14] reported that the basal rate of soil respiration was markedly affected by the spatial variability of soil P (*p* = 0.04) among nine broadleaf forest stands. The possible reason is that available P is the essential nutrient element for growth of plants and soil microorganisms. Therefore, the role of soil P as an important determiner of soil respiration rate cannot be ignored. Nonetheless, we did not observe a significant correlation between the annual mean soil respiration rate and the C/N ratio, pH, available K, or water soluble Al.

### Relationship of soil respiration with soil temperature and moisture

**Fig 7:**
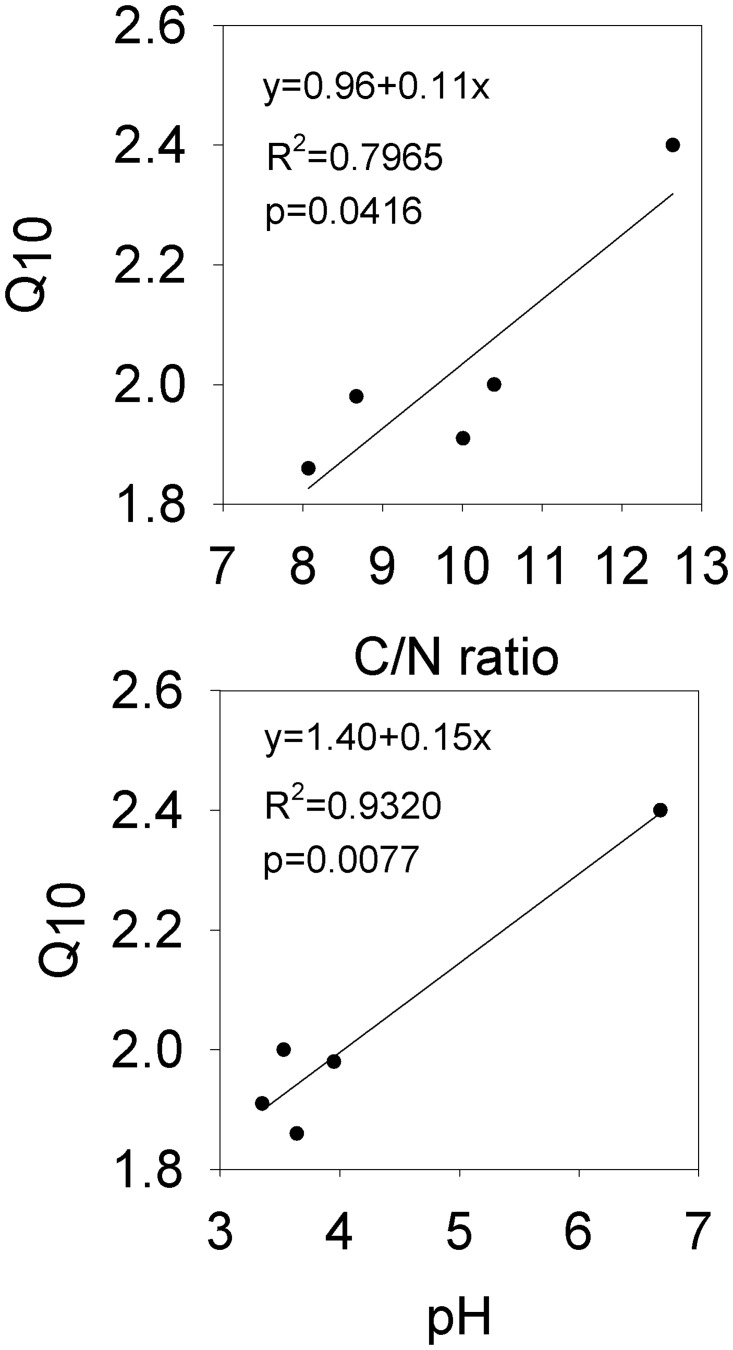
Correlation of Q10 with the C/N ratio and pH.

**Fig 8:**
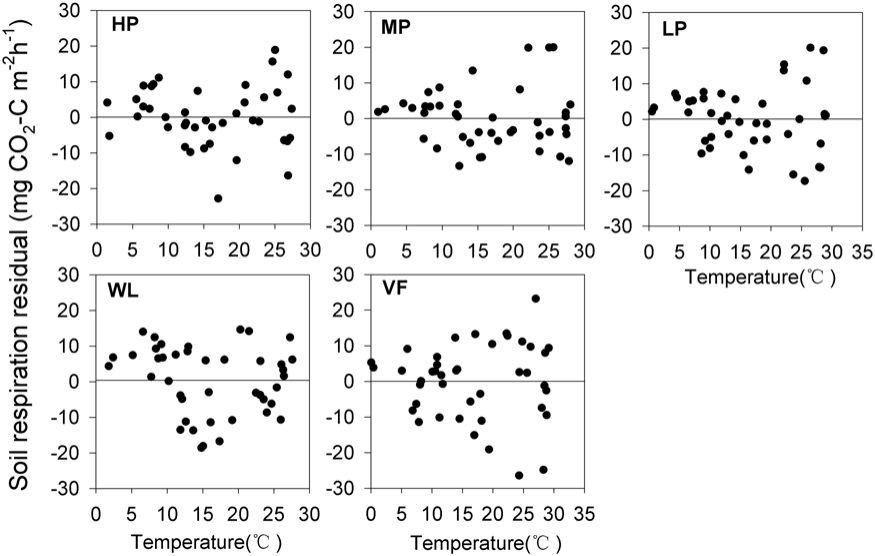
Variation of the soil respiration residual with temperature. HP = tea garden with high production, MP = tea garden with medium production, LP = tea garden with low production, WL = woodland, VF = vegetable field.

Numerous studies have shown seasonal variation of the soil respiration rate with a single peak, consistent with the variation tendency of soil temperature [45], [46]. We found this same trend, and the peak and trough of this single peak at each site occurred almost at the same time (Fig 2). Similarly, we observed a strong exponential relationship between the soil respiration rate and soil temperature at each site, and the relationship with temperature measured at 5 cm soil depth was stronger than that measured at the soil surface (Fig 6). Up to 87–93% of the variation of soil respiration was explained by the soil temperature at 5 cm depth, more than the 77–88% at the surface, because the temperature at the surface was unstable and more easily affected by air circulation, illumination, and vegetation cover [47]. Furthermore, the Q_10_ value calculated by soil temperature measured at 5 cm depth and the R^2^ were higher than those based on temperature measured at the soil surface at each site (Fig 2). We therefore selected the Q_10_ calculated based on the temperature at 5 cm soil depth. Furthermore, our results revealed that converting woodland to a tea garden decreased Q_10_, whereas woodland converted to the vegetable field increased the Q_10_ value. These results clearly indicate the negative effect of land-use change on the vegetable field, and the positive effect of the conversion to tea garden as a better adaption to global climate warming. The Q_10_ values that vary between the different vegetation would also be affected by the factors that influence the soil respiration rate. We found that Q_10_ is significantly related to the C/N ratio and pH (Fig 7), and positively correlated with organic C, total N, and available P and K. Zhou *et al*.[14] also found that Q_10_ was influenced by ecological factors such as temperature, soil moisture, microbial biomass, and vegetation properties and types, all of which mainly depend on the control of substrate availability in operation. The Q_10_ value would change with increasing temperature [48], [49]. In other words, if we use Q_10_ as a fixed value to compute the exponential model, the accuracy of the simulation values would reduce with the increasing temperature. We found strong evidence of this in our study (Fig 8); when temperature was low, the residual values (obtained by subtracting the calculated values from the observed) were closed on both delta neutral and delta sides, and their discrete degree increased with temperature. Therefore, accurate quantification of the Q_10_ parameter values is essential for a reliable estimate of the ecosystem carbon balance [50] and further studies are required.

The effect of soil moisture on soil respiration observed in our study was small, which was consistent with Davidson *et al*. [27], who studied the relationship between soil respiration and soil moisture in a temperate mixed forest using Eq 3, and Sotta *et al*. [28] who studied an amazon rainforest based on Eq 4. However, Davidson *et al*. [27] found significant correlation between soil respiration and moisture when the soil volumetric water content was less than 12%. Similarly, Cui *et al*. [51] found a highly significant correlation between soil respiration and moisture in a semi-arid area of Inner Mongolia, China, that received light rainfall during a major portion of the year. We therefore suggest a mechanism where soil structure and composition, such as soil bulk density, particle composition, pore geometry, and distribution, can affect the moisture status of soil. The moisture status of dry periods is conducive to the diffusion of oxygen, but not to the substrate supply. When soil moisture is low, the connection of soil particles will be reduced by excess air and, at the same time, the transport of nutrients is blocked, limiting soil microbial growth and activities, and soil moisture will increase as the limiting factor for soil respiration. The moisture status of the lower matric potential is conducive to the diffusion of substrate, but not to oxygen supply. Furthermore, the lower matric potential in the surrounding soil inhibits the diffusivity of solutes between microbe cells and the outside solution, and the anoxic environment reduces microbial growth and activity. Plants also need available water as both drought and waterlogging hinder plant growth and root respiration. In addition, the two-variable model combining soil temperature and moisture was better at predicting the seasonal variation of the soil respiration rate [52], [53], and this relationship was observed at each site in our study (Table 2).

### Driving factors of soil respiration and Q_10_ under different land uses

The annual mean soil respiration rate and Q_10_ were driven by the identical factors of SWC, SOC, pH, and WSAl following the land use conversion. SOC was positively correlated with both the annual mean soil respiration rate and Q_10_. However, SWC, pH, and WSAl were negatively correlated with the annual mean soil respiration rate and positively correlated with Q_10_. It was essential to include SOC in the diagnostic factors and, as expected, an increase in SOC could also increase the annual mean soil respiration rate (Fig 5a). Davidson *et al*. [32] found that Q_10_ increased with the increase of activation energy under a given temperature, which is in agreement with our current observations. Based on the above discussion, we can assume that the effect of soil water content in driving soil respiration was underestimated. Markedly, there were no differences in soil temperature between the five sites; however, the SWC values were fairly different (Table 1). Our results are in accordance with the report of Fóti *et al*. [22], who found that the spatial range of the soil respiration rate was negatively correlated (*p* < 0.01) with the transect average of SWC in a semi-arid sandy grassland. Other previous studies have also shown that soil respiration remained stable under a certain range of water content, which would tend to decrease when water content exceeds this range under a controlled temperature [20], [54]. Therefore, soil moisture can mask the effect of soil temperature in controlling the soil respiration rate at the spatial scale to some extent. Furthermore, Davidson *et al*. [27] demonstrated that a well-drained site had a lower Q_10_ than a poorly-drained site in a temperate mixed hardwood forest. Q_10_ values of 1.4 and 1.8 were recorded in a young ponderosa pine plantation in northern California with a soil moisture of <14% and >14%, respectively [55]. It is now clear from the above description that Q_10_ was positively correlated with SWC. Although the simple linear relationship between the annual mean soil respiration rate and pH or WSAl, and between Q_10_ and WSAl was not significant, pH and WSA1 were included in the final equation because long-term tea cultivation with heavy nitrogen application and leaf litter containing a large amount of aluminum may result in the decline of pH and accumulation of Al in tea garden soils.

## Conclusions

In summary, by determining the annual variation of the soil respiration rate in five adjacent sites (HP, MP, LP, WL, and VF) in Eastern China, we found that the soil respiration dynamics in a tea garden, woodland, and vegetable field all showed a single peak curve, and an increase in the annual cumulative respiration flux when woodland was converted to a high production tea garden or vegetable field. The increase in the annual mean soil respiration rate was coupled with an increase in organic C, total N, and available P. Soil temperature at 5 cm depth can better simulate the relationship between the soil respiration rate and temperature than the temperature at the soil surface. The model with a combination of soil temperature and moisture could better predict the temporal variation of the soil respiration rate than the single temperature model for all sites. Q_10_ increased when woodland was converted to a vegetable field, but decreased when converted to tea gardens. Q_10_ was significantly and positively correlated with the C/N ratio and pH value, which indicates that both the C/N ratio and pH are indexes that reflect the difference of Q_10_ between different ecosystems. SWC, SOC, pH, and WSAl drove the changes in soil respiration and Q_10_ following the conversion of land use. Overall, temporal variations of soil respiration were mainly controlled by soil temperature, and spatial variations were influenced by SOC, SWC, pH, and WSAl. The results of this investigation have important implications for studying soil respiration in a woodland ecosystem that has been converted to a tea garden or vegetable field in Eastern China.
